# Degradation kinetics and protein intestinal digestibility of tomato pomace in goats

**DOI:** 10.3389/fvets.2025.1724783

**Published:** 2026-01-09

**Authors:** Yong Long, Siwaporn Paengkoum, Shengyong Lu, Xinran Niu, Sorasak Thongpea, Nittaya Taethaisong, Yong Han, Pramote Paengkoum

**Affiliations:** 1 School of Animal Technology and Innovation, Institute of Agricultural Technology, Suranaree University of Technology, Nakhon Ratchasima, Thailand; 2 Guizhou University of Traditional Chinese Medicine, Guiyang, China; 3 Program in Agriculture, Faculty of Science and Technology, Nakhon Ratchasima Rajabhat University, Nakhon Ratchasima, Thailand

**Keywords:** degradation kinetic parameters, intestinal digestibility, ruminally cannulated goats, three-step *in vitro* procedure, tomato pomace

## Abstract

**Introduction:**

This study aimed to analyze and compare the degradation kinetics and intestinal protein digestibility of soybean meal (SBM), whole tomato (WT), and tomato pomace (TP) in the rumen of goats.

**Methods:**

Three Boer goats were fitted with permanent rumen cannulas, and all samples were incubated *in situ* in the rumen for 2, 4, 8, 12, 24, 48, and 72 h using the nylon bag technique. Six nylon bags were prepared for each ruminally cannulated goat in each period (*n* = 6), resulting in a total of 18 nylon bags for the three goats. Additionally, the samples prepared in the same way were removed following 16 h of *in situ* incubation in the rumen, the *in vitro* three-step procedure was used to analyze intestinal protein digestibility.

**Results:**

The results showed that the degradation rates of dry matter (DM), organic matter (OM), crude protein (CP), neutral detergent fiber (NDF), and acid detergent fiber (ADF), as well as the effective degradability (ED) and a* values in WT were higher (*P* < 0.01) than those in SBM. Moreover, the degradation rates of DM and OM in TP were higher (*P* < 0.01) than those in SBM (except at 72h of incubation), whereas CP degradation in TP surpassed that of SBM (*P* < 0.01) only following 8 h of incubation. Finally, the small intestinal digestibility of CP (Idg, %) and small intestine digests CP (IDCP, g/kg) of WT and TP were higher (*P* < 0.01) than those of SBM.

**Conclusion:**

WT and TP exhibit a higher degradation rate in the rumen and better protein digestibility in the intestine.

## 1 Introduction

Tomatoes (*Lycopersicon esculentum*), one of the most popular vegetables worldwide, are celebrated for their high content of beneficial compounds, including lycopene, vitamins, phenols, organic acids, and crude protein (CP) (1, 2). Apart from being a typical vegetable, tomatoes undergo processing into purees, pastes, juices, and sauces, leading to the creation of a byproduct referred to as tomato pomace (TP) (3, 4). TP is mainly composed of seeds, peel, and a small fraction of pulp, which collectively account for about 3–5% of the whole tomato (WT) (5). As of 2021, tomato production has reached 189 million tons, while TP production exceeds 39 million tons in the world (6, 7). Although TP is rich in nutrients, its high moisture content (about 75%) makes it particularly susceptible to bacterial contamination and spoilage. Improper disposal not only results in the waste of valuable resources but also poses serious environmental pollution risks. As a result, finding pollution-free treatment and utilization solutions has become an urgent issue for the food industry (8, 9). Importantly, TP is rich in lycopene, tocopherol, β-carotene, terpenes, and polyphenols, which are bioactive compounds with antioxidant properties (9, 10). The rational utilization of TP is a valuable resource that can extract lycopene, tomato seed oil, and dietary fiber (2, 11).

The development of sustainable animal feeds utilizing agricultural by-products has emerged as a critical research frontier in nutritional science, driven by the triple imperative of mitigating environmental impacts, optimizing production costs, and alleviating food-feed competition (12–15). Within this context, tomato pomace (TP) demonstrates substantial potential as a novel ruminant feed resource (16). Bureenok et al. (17) concluded that supplementing Leucaena silage with dried TP at levels of 0, 0.2, 0.4, and 0.8% in diets of male crossbred Anglo-Nubian goats enhanced the digestibility of dry matter (DM), organic matter (OM), CP, neutral detergent fiber (NDF), and acid detergent fiber (ADF). Moreover, a positive relationship was observed between the extent of supplementation and the degree of improvement. Similarly, Mizael et al. (18) found that including TP at 20, 40, and 60% in the diets of Saanen dairy goats significantly enhanced the apparent digestibility of CP, ether extract (EE), and Non-fibrous Carbohydrates (NFC). Furthermore, Abd-Elkerem et al. (19) demonstrated via *in vitro* gas production that exogenous enzyme treatment of tomatoes enhanced the degradation of DM, CP, and crude fiber (CF). Differently, Bakshi et al. (20) observed that the inclusion of TP in the total mixed ration of male Murrah buffaloes reduced CP content while increasing hemicellulose. However, no significant influence was detected on daily DM intake or the digestibility of the remaining nutrients. On the other hand, according to Omer et al. (21), the supplementation of 5, 10%, or 15% dried TP in the total mixed ration of male Ossimi lambs did not affect CP or DM digestibility. However, supplementation at 10 and 15% significantly enhanced the digestibility of OM, CF, EE, and nitrogen-free extract, as well as the total digestible nutrients value. Surprisingly, Robles-Jimenez et al. (22) reported that supplementing corn silage-based diets of Suffolk lambs with 100 or 200 g/kg of dry whole green tomatoes negatively affected nutrient digestibility and nitrogen balance.

Current research on the digestibility and ruminal degradation of TP in ruminants remains limited, and scholarly consensus has yet to be reached, with varying conclusions reported. However, research into TP degradation kinetics in the rumen and digestibility in the small intestine is even rarer. We hypothesize that TP may be more digestible, absorbable, and usable in goats compared to soybean meal (SBM). Therefore, this study will use the nylon bag technique and the *in vitro* three-step method to clearly clarify the degradation kinetic parameters and intestinal digestibility of TP in the rumen of goats, to provide a theoretical basis for the development of TP feed.

## 2 Materials and methods

### 2.1 Feed samples and preparation

Tomatoes were purchased from the agricultural products market in Nakhon Ratchasima, Thailand. The WT sample in this experiment was sliced and dried directly from the purchased tomato. The TP sample was made from the peel and seeds that are left over after juice and paste of purchased tomatoes. SBM samples are provided by the farm of the School of Animal Technology and Innovation, Institute of Agricultural Technology, Suranaree University of Technology (SUT). All samples were dried at 65 °C, ground, and then passed through a 2 mm screen before being stored for subsequent nylon bag technology studies and chemical analysis. The approximate nutritional composition of tomatoes and TP is shown in Table 1.

**Table 1:** Approximate nutritional composition and lycopene content of WT and TP.

Items	Moisture %	DM %	CP %	EE %	NDF %	ADF %	ADL %	Ash %	Lycopene (μg/g)
WT	93.55	87.65	11.54	8.11	44.57	16.70	2.72	11.00	75.98
TP	89.19	88.99	16.05	12.21	51.25	24.00	3.68	10.08	63.61
SBM	9.23	89.18	46.24	1.56	7.87	4.69	0.52	6.24	–

WT, whole tomato; TP, tomato pomace; SBM, soybean meal; Moisture, initial moisture content, ADL, acid detergent lignin. Ash, crude ash.

All nutritional composition analyses except moisture parameters were analyzed on a DM basis.

### 2.2 Animals, management, and experimental design

Three rumen-cannulated Boer goats (24.1 ± 1.3 kg) were kept in a 3 × 5 m enclosure. The experiment followed a completely randomized design (CRD) with three feed ingredients (WT, TP, and SBM) incubated simultaneously in the rumen of the three goats, which served as biological replicates (*n* = 3 goats). Feed was offered twice daily at 08:30 and 16:30, with free access to water. The goats were fed a basal diet ration equivalent to 4% of body weight. The formulation of the basal diet was based on the nutrient requirements specified by NRC (23), and its chemical composition is presented in Table 2. The experiment consisted of a 15-day adaptation period followed by a 7-day sample collection period.

**Table 2:** Feed ingredients and nutrient components (%, air-dry basis).

Ingredient	Contents, %
Soybean meal	3.75
Soybean hulls	3.6
Corn seed	3
Rice bran	3.75
Molasses	0.45
Calcium phosphorus	0.15
Salt	0.15
Premix^1^	0.15
Silage corn	85
Total	100
Chemical composition, %^2^	
DM	35.35
CP	13.68
Ash	10.01
NDF	59.14
ADF	34.24
Ca	0.55
P	0.48
Metabolizable energy (ME), MJ/kg	11.72

^1^ Contains per kilogram premix: 10,000,000 IU vitamin A; 70,000 IU vitamin E; 1,600,000 IU vitamin D; 50 g iron; 40 g zinc; 40 g manganese; 0.1 g cobalt; 10 g copper; 0.1 g selenium; 0.5 g iodine.

^2^ Except for the energy value, the remaining chemical components are actual measured values.

### 2.3 *In situ* ruminal degradation procedure

The degradation kinetics of CP, OM, DM, NDF, and ADF were employed for ruminal in-situ fermentation and were determined by the methods of Dong et al. (24) and Ørskov and McDonald (25). Nylon bags (6 × 12 cm, 40 ± 5 μm pore size) were filled with 10 g (air-dry basis) of each sample (surface area ratio < 20 mg/cm^2^). Bags were heat-sealed and tied to a 40-cm nylon line attached to the cannula cap.

Incubations were performed sequentially with reverse timing so that all bags were removed simultaneously at 08:00 h on the final day (standard procedure to minimize diurnal variation). Incubation times were 2, 4, 8, 12, 24, 48, and 72 h. In each incubation period, 6 nylon bags (*n* = 6) were prepared for each ruminally cannulated goat, resulting in a total of 18 bags for the three goats. Across all 7 incubation periods, 126 nylon bags were prepared in total. Zero-hour bags (*n* = 18 per feedstuff) were not incubated in the rumen but were washed identically to incubated bags.

After the incubation time for each stage, the nylon bag was removed from the rumen and washed in a washing machine for 10 min until the solution became neutral. Samples were dried at 60 °C for 48 h and subsequently ground to pass through a 2 mm sieve. The nylon bag incubated for 0 h is a control bag, and it was used using the identical process as the bags that were incubated (25). Finally, after all procedures were completed, the six samples from each goat at different incubation time points were stored. For chemical analysis, each sample from each period was analyzed in three replicates, and the average result was used.

### 2.4 *In vitro* three-step procedure

The *in vitro* three-step method was improved by referring to the method of Boucher et al. (26). The same method was used to accurately weigh 10 g samples, which were then incubated in the rumen of cannula goats for 16 h. After removal from the rumen, the nylon bag was suspended in a 0.1% methylcellulose solution and incubated at 37 °C for 30 min. Afterward, the sample was removed and placed in a refrigerator at −20 °C for storage. Before the gastric protease assay, nylon bags were thawed and subjected to three successive washes in a washing machine, each lasting 5 min, in order to remove residual rumen bacteria. The bags were subsequently dried in an oven at 65 °C and passed through a 1 mm sieve. Enzymes, including pepsin (P-7000) and trypsin (P-7545), were obtained from Sigma (USA). Subsequently, 1 g of undegraded rumen residue was weighed into a nylon bag, which was immersed in 10 mL of solution adjusted to pH 1.9 with 0.1 N HCl and supplemented with 1 g/L pepsin (Sigma P-7000). The incubation was performed in a shaking water bath at 39 °C for 1 h with intermittent vortexing. After the incubation was completed, 0.5 mL of 1 N NaOH solution and 13.5 mL of trypsin solution (0.5 M KH_2_PO_4_ buffer, adjusted to pH 7.75, containing 3 g/L trypsin, Sigma P-7545, Sigma) were supplemented. The incubation of the samples was carried out in a shaking water bath maintained at 39 °C for a period of 24 h, during which vortexing was applied at 4 h intervals. Upon completion of the incubation, the bags were washed repeatedly with tap water until neutrality of the rinsing solution was reached, followed by drying at 65 °C until a stable weight was obtained (about 48 h). Next, the samples were weighed on an analytical balance, and the results were documented. The CP content of the samples collected in the bags was subsequently analyzed in the laboratory.

### 2.5 Chemical analysis

In the laboratory, the dried samples underwent compositional analysis to determine CP (No. 976.05), DM (No. 934.01), EE (No. 973.18), and Ash (No. 942.05) according to the AOAC (27). The contents of NDF and ADF were assessed using the procedure of Van Soest et al. (28), where sodium sulfite was incorporated, thermostable α-amylase was omitted, and the NDF values were reported exclusive of residual Ash. The value of OM was calculated by subtracting Ash from DM. Additionally, high-performance liquid chromatography (HPLC, 1200, Agilent Technologies, USA) was used to analyze the lycopene content in WT and TP. All procedures for lycopene extraction and analysis, including the addition of ethyl acetate, methyl tert-butyl ether, and methanol, as well as the HPLC analytical procedures, were strictly adhered to as outlined by Li et al. (29).

### 2.6 Fatty acid and amino acid profiles

Fatty acid profiles were determined to provide a comprehensive nutritional characterization of the novel tomato by-products, which may be relevant for precision feeding studies. Total lipids were extracted and methylated following Tian et al. (30). Gas chromatography (GC–MS, SQ8T, PerkinElmer, USA) was used to analyze the samples with n-hexane as the internal standard. A total of 37 fatty acids were subsequently quantified.

Amino acid profiles were included for the same purpose (complete nutritional evaluation). WT and TP samples were dried and ground prior to analysis. Approximately 100 mg of material was transferred into a 20 mL glass hydrolysis tube, to which 10 mL of 6.0 mol/L hydrochloric acid was added. The mixture was thoroughly shaken and evacuated under vacuum, and the tubes were then sealed. Hydrolysis was carried out in a constant-temperature oven at 110 ± 2 °C for 22 h. Following hydrolysis, the tubes were removed and cooled to ambient temperature. Once cooled, the pH was neutralized with 6.0 mol/L sodium hydroxide, and the volume was adjusted to 25 mL with distilled water. Furthermore, accurately pipette 1.0 mL of the filtrate and transfer it to a 15 mL test tube for decompression treatment, and then place it in a constant-temperature oven set to 45 °C. After drying, a small amount of ultrapure water was used to dissolve the residue, and the above drying operation was repeated 1–2 times. Afterward, the mixed amino acid standard and sample solutions were injected into the amino acid analyzer (Hitachi L-8900, Japan) at a 1:1 ratio. The concentrations of amino acids in the sample were calculated using the peak area from the external standard method.

### 2.7 Kinetic modeling and statistical analysis

The disappearance rates of DM and CP from nylon bags were modeled using the exponential equation proposed by Ørskov and McDonald (25), fitted through non-linear regression procedures (SAS, 9.4):


$$P=a+b\;(1-e^{–\mathrm{ct}})$$

In this model, P represents nutrient disappearance at incubation time t. The parameter a denotes the soluble fraction that is rapidly washed out of the bags and is considered fully degradable. The parameter b corresponds to the insoluble fraction that can potentially be degraded by microorganisms. The constant e refers to the base of the natural logarithm, ccc is the degradation rate constant of fraction b per hour (i.e., k), and t indicates the incubation period.

The *in situ* effective degradability (ED) of DM and CP for each feed sample in the two lambs was calculated according to the following equation (31):


ED=a+(b×c)/(k+c)

In this expression, ED represents the effective degradation rate (%), while k denotes the ruminal passage rate of feed, fixed at 0.05%/h.

The effective degradability of CP is commonly expressed as the rumen-degradable protein (RDP) fraction of CP in feed, while the undegraded portion is defined as rumen-undegradable protein (RUP). The calculation is presented as follows (31):


RDP(g/kg)=CP(%)×ED/10

RUP(g/kg)=CP(%)×10−RDP

The small intestinal digestibility of CP (Idg, %) of RUP and the small intestine digests CP (IDCP, g/kg) in the small intestine are as follows (31):


$$\mathrm{Idg}\;(\%)=100\times ({\mathrm{CP}}_{16h}-{\mathrm{CP}}_{N})/{\mathrm{CP}}_{16h}$$

IDCP(g/kg)=RDP×0.775×0.85+RUP×Idg

where CP_16h_ denotes the protein content of rumen-degraded residues after 16 h of incubation (g/kg), and CP_N_ refers to the protein remaining after small-intestinal digestion (g/kg). The degradation coefficient of microbial crude protein (MCP) in the rumen was fixed at 0.85, and the coefficient for MCP digestibility in the small intestine was 0.775 (23).

Values of a, b, and c, together with ED and the outcomes of the three-step *in vitro* procedure, were analyzed using the GLM procedure of SAS according to the following model:


Yij=μ+dij+eij

where Yij represents the trait under investigation, μ the overall mean, dij the effect of feed source, and eij the residual error.

Data analysis was performed by ANOVA in SAS (version 9.4), and significant differences among means were identified using Duncan’s multiple-range test. All values are presented as means with their standard errors (SEM), and statistical significance was considered at *p* < 0.05.

## 3 Results

### 3.1 Fatty acid composition of WT and TP

From Table 3, it can be concluded that TFA in TP is higher than that in WT. The fatty acids in WT and TP were primarily C18:2 n-6, C18:3 n-3, C18:1 n-9, C18:0, and C16:0, with C18:2 n-6 exhibiting the highest content, followed by C16:0. The concentrations of SFA, PUFA, MUFA, and UFA in TP were higher than those in WT. Interestingly, the *Σ* n-6/Σ n-3 ratio of TP was lower than that of WT.

**Table 3:** Fatty acid composition of WT and TP (g/100 g).

Items	WT	TP
C8:0	0.03	0.04
C10:0	0.02	0.04
C12:0	0.12	0.19
C14:0	0.30	0.42
C15:0	0.05	0.09
C16:0	18.72	21.15
C16:1	0.19	0.24
C17:0	0.11	0.19
C18:0	4.11	6.85
C18:1 n-9	8.62	10.33
C18:2 n-6	31.95	34.29
C18:3 n-3	9.25	11.62
C20:0	0.41	0.61
C20:1	0.03	0.17
C20:2	0.02	0.06
C20:3 n-3	0.18	0.23
C20:4 n-6	1.15	1.65
C20:5 n-3	0.01	0.07
C22:2	0.03	0.31
C23:0	0.45	0.47
C24:0	0.24	0.36
SFA	24.55	30.42
MUFA	8.84	10.74
PUFA	42.59	48.24
UFA	51.43	58.98
TFA	75.98	89.40
Σ n-3	9.44	11.93
Σ n-6	33.10	35.94
Σ n-6/*Σ* n-3	3.51	3.01

SFA, saturated fatty acids; MUFA, monounsaturated fatty acids; PUFA, polyunsaturated fatty acids; UFA, unsaturated fatty acids; TFA, Total fatty acids; Σ n-3, sum of n-3 fatty acids; Σ n-6, sum of n-6 fatty acids.

### 3.2 Amino acid composition of WT and TP

As shown in Table 4, TP contained higher concentrations of EAA, NEAA, and TAA compared with WT. With the exception of glutamic acid, all amino acid levels in WT were lower than those observed in TP.

**Table 4:** Amino acid composition of WT and TP (g/100 g).

Items	WT	TP
Aspartic acid	1.71	1.81
Threonine	0.21	0.34
Serine	0.33	0.48
Glutamic acid	4.84	4.16
Glycine	0.31	0.45
Alanine	0.40	0.41
Valine	0.19	0.27
Isoleucine	0.22	0.27
Leucine	0.41	0.51
Tyrosine	0.17	0.31
Phenylalanine	0.22	0.31
Histidine	0.11	0.13
Lysine	0.20	0.29
Arginine	0.42	0.43
Methionine	0.02	0.08
EAA ^1^	1.84	2.54
NEAA^2^	7.62	7.74
TAA^3^	9.46	10.28
FAA	7.67	7.26

^1^ EAA, essential amino acids (Threonine, Alanine, Isoleucine, Leucine, Phenylalanine, Histidine, Lysine, Arginine, Methionine).

^2^ NEAA, non-essential amino acids (Aspartic acid, Serine, Glutamic acid, Glycine, Alanine, Tyrosine).

^3^ TAA, total amino acids.

FAA, flavor amino acids (Alanine, Glycine, Arginine, Glutamic acid, Aspartic acid).

### 3.3 Effects of different feed materials on the degradability of DM

Table 5 illustrates that the degradation rate of DM in all groups rose progressively as the duration of rumen *in situ* incubation was prolonged. The degradation rates of DM in WT and TP at 2, 4, 8, 12, 24, and 48 h were higher (*p* < 0.01) than those in the SBM group. Additionally, the DM degradation rate in WT was higher (*p* < 0.01) than that in TP at 2, 4, 8, and 12 h. The values of a^*^ and effective degradation (ED) rates of WT and TP were higher (*p* < 0.01) than those of SBM. However, the b^*^ values of SBM were the opposite (*p* < 0.01).

**Table 5:** Effect of different treatments on DM degradability (%).

Items	SBM	WT	TP	SEM	*p*-value
2 h	34.47^c^	70.60^a^	51.57^b^	3.51	<0.01
4 h	37.80^c^	72.80^a^	56.57^b^	4.35	<0.01
8 h	43.73^c^	76.53^a^	63.97^b^	4.08	<0.01
12 h	48.80^c^	79.47^a^	69.57^b^	3.77	<0.01
24 h	60.13^b^	85.07^a^	78.20^a^	3.91	<0.01
48 h	72.50^b^	89.50^a^	84.40^a^	2.71	<0.01
72 h	82.27^b^	90.90^a^	86.43^ab^	2.33	0.036
a^*^	30.90^c^	68.10^a^	46.2^b^	2.88	0.013
b^*^	55.57^a^	23.83^c^	44.10^b^	2.51	<0.01
c^*^	0.04	0.05	0.06	0.01	0.631
a + b^*^	91.93	90.30	89.00	2.96	0.940
ED	64.83^b^	80.30^a^	69.93^a^	1.67	<0.01

SBM, soybean meal; WT, whole tomato; TP, tomato pomace.

a^*^, the soluble nutrient fraction rapidly washed out of the bags and assumed to be completely degradable; b^*^, the proportion of insoluble nutrients potentially degradable by microorganisms; c^*^, the degradation rate of fraction b^*^ per hour; a + b^*^, potential degradability; ED, effective degradability.

Different superscript letters within the same row indicate significant differences in the data for this parameter (*p* < 0.05). The descriptions for Tables 6–9 follow the same format in this study.

### 3.4 Effects of different feed materials on the degradability of OM

The degradation rates of OM are summarized in Table 6. Before 48 h, the OM degradability of WT was significantly lower (*p* < 0.01) than that of TP, whereas the b^*^ value showed the opposite pattern (*p* < 0.01). Interestingly, the a^*^ values of WT and TP were significantly higher (*p* < 0.01) than those in SBM, with the highest a^*^ value for OM in WT. The ED value of SBM was the lowest (*p* < 0.05).

**Table 6:** Effect of different treatments on OM degradability (%).

Items	SBM	WT	TP	SEM	*p*-value
2 h	32.53^c^	66.70^a^	48.57^b^	4.94	<0.01
4 h	38.20^c^	70.43^a^	55.47^b^	4.69	<0.01
8 h	43.20^c^	75.07^a^	63.57^b^	4.1	<0.01
12 h	47.77^c^	76.57^a^	68.33^b^	4.67	<0.01
24 h	57.93^b^	86.63^a^	75.60^a^	4.50	<0.01
48 h	68.60^b^	83.73^a^	79.33^a^	2.71	<0.01
72 h	81.07^b^	89.50^a^	82.00^ab^	1.51	0.03
a^*^	30.97^c^	59.90^a^	42.20^b^	4.28	<0.01
b^*^	53.83^a^	27.70^c^	42.03^b^	3.01	<0.01
c^*^	0.05	0.05	0.06	0.01	0.686
a + b^*^	84.80	87.60	84.80	1.02	0.407
ED	60.77^b^	69.30^a^	66.53^a^	1.48	0.022

SBM, soybean meal; WT, whole tomato; TP, tomato pomace.

a^*^, the soluble nutrient fraction rapidly washed out of the bags and assumed to be completely degradable; b^*^, the proportion of insoluble nutrients potentially degradable by microorganisms; c^*^, the degradation rate of fraction b^*^ per hour; a + b^*^, potential degradability; ED, effective degradability.

Different superscript letters within the same row indicate significant differences in the data for this parameter (*p* < 0.05).

### 3.5 Effects of different feed materials on the degradability of CP

As shown in Table 7, before 12 h, the CP degradability of WT was the highest (*p* < 0.01), and at 12 h, the CP degradability in TP was higher (*p* < 0.01) than that in SBM and WT. Compared with the SBM, WT, and TP exhibited higher (*p* < 0.01) CP degradability at 24, 48, and 72 h, and the same pattern was observed for their b^*^ and a + b^*^ values (*p* < 0.01). The ED in the WT group was improved (*p* < 0.01) compared with SBM.

**Table 7:** Effect of different treatments on CP degradability (%).

Items	SBM	WT	TP	SEM	*p*-value
2 h	20.10^b^	27.20^a^	20.37^b^	1.20	<0.01
4 h	24.83^b^	34.87^a^	28.43^b^	1.55	<0.01
8 h	30.77^c^	45.40^a^	37.63^b^	2.13	<0.01
12 h	37.30^c^	47.20^b^	53.40^a^	2.40	<0.01
24 h	47.03^b^	67.83^a^	63.20^a^	3.28	<0.01
48 h	55.17^b^	74.83^a^	68.60^a^	2.98	<0.01
72 h	59.73^b^	76.98^a^	74.27^a^	2.85	<0.01
a^*^	18.17	20.10	18.33	0.56	0.405
b^*^	38.5^b^	52.20^a^	52.0^a^	2.40	<0.01
c^*^	0.03	0.04	0.05	0.01	0.771
a + b^*^	56.67^b^	72.30^a^	71.33^a^	2.53	<0.01
ED	52.07^b^	60.27^a^	56.90^ab^	1.28	<0.01

SBM, soybean meal; WT, whole tomato; TP, tomato pomace.

a^*^, the soluble nutrient fraction rapidly washed out of the bags and assumed to be completely degradable; b^*^, the proportion of insoluble nutrients potentially degradable by microorganisms; c^*^, the degradation rate of fraction b^*^ per hour; a + b^*^, potential degradability; ED, effective degradability.

Different superscript letters within the same row indicate significant differences in the data for this parameter (*p* < 0.05).

### 3.6 Effects of different feed materials on the degradability of NDF

In Table 8, at 2, 4, 24, 48, and 72 h, the NDF degradability of WT was higher (*p* < 0.05) than that of both SBM and TP. Compared with the SBM, the WT showed increased (*p* < 0.05) a^*^, b^*^, a + b^*^, and ED values.

**Table 8:** Effect of different treatments on NDF degradability (%).

Items	SBM	WT	TP	SEM	*p*-value
2 h	17.40^b^	24.53^a^	17.47^b^	1.21	<0.01
4 h	22.93^b^	29.43^a^	23.33^b^	1.15	<0.01
8 h	30.87^b^	37.23^a^	32.63^ab^	1.57	0.047
12 h	38.67	45.00	39.33	2.07	0.446
24 h	46.63^b^	53.17^a^	46.70^b^	1.73	0.045
48 h	51.20^b^	60.03^a^	50.30^b^	1.74	<0.01
72 h	55.43^b^	62.4^a^	55.03^b^	1.75	0.048
a^*^	15.00^b^	23.07^a^	18.83ab	0.70	<0.01
b^*^	35.73^b^	40.70^a^	37.27^b^	0.82	<0.01
c^*^	0.04	0.04	0.03	0.01	0.912
a + b^*^	50.73^c^	62.90^a^	56.63^b^	1.80	<0.01
ED	39.70^b^	53.33^a^	40.73^ab^	2.07	<0.01

SBM, soybean meal; WT, whole tomato; TP, tomato pomace.

a^*^, the soluble nutrient fraction rapidly washed out of the bags and assumed to be completely degradable; b^*^, the proportion of insoluble nutrients potentially degradable by microorganisms; c^*^, the degradation rate of fraction b^*^ per hour; a + b^*^, potential degradability; ED, effective degradability.

Different superscript letters within the same row indicate significant differences in the data for this parameter (*p* < 0.05).

### 3.7 Effects of different feed materials on the degradability of ADF

In Table 9, at all rumen *in situ* incubation times tested, the ADF degradation rate of SBM was lower (*p* < 0.01) than that of WT at 4, 8, 12, 24, and 48 h, but no significant difference was observed between SBM and TP (*p* > 0.05). The a^*^, b^*^, a + b^*^, and ED values of the WT group were higher (*p* < 0.01) than those of the SBM and TP. On the other hand, compared with SBM, the a^*^ value of TP was also increased (*p* < 0.01).

**Table 9:** Effect of different treatments on ADF degradability (%).

Items	SBM	WT	TP	SEM	*p*-value
2 h	6.93^b^	11.57^a^	8.23^b^	0.80	0.018
4 h	9.03^b^	13.77^a^	13.07^a^	0.81	<0.01
8 h	12.87^b^	18.97^a^	17.20 ^ab^	1.02	<0.01
12 h	15.90^b^	23.67^a^	19.47^ab^	5.64	<0.01
24 h	24.87^b^	30.17^a^	25.57 ^ab^	1.12	0.047
48 h	29.97^b^	36.23^a^	29.57^b^	1.12	<0.01
72 h	32.53^b^	42.57^a^	32.27^b^	1.75	<0.01
a^*^	4.60^c^	13.40^a^	7.50^b^	0.18	<0.01
b^*^	26.47^b^	33.17^a^	22.90^b^	1.57	<0.01
c^*^	0.04	0.04	0.03	0.00	0.282
a + b^*^	31.07^b^	46.57^a^	30.40^b^	2.69	<0.01
ED	25.47^b^	29.47^a^	25.53^b^	0.72	<0.01

SBM, soybean meal; WT, whole tomato; TP, tomato pomace.

a^*^, the soluble nutrient fraction rapidly washed out of the bags and assumed to be completely degradable; b^*^, the proportion of insoluble nutrients potentially degradable by microorganisms; c^*^, the degradation rate of fraction b* per hour; a + b^*^, potential degradability; ED, effective degradability.

Different superscript letters within the same row indicate significant differences in the data for this parameter (*p* < 0.05).

### 3.8 Trend diagram of degradation curves of nutritional parameters of different feed materials

**Figure 1:**
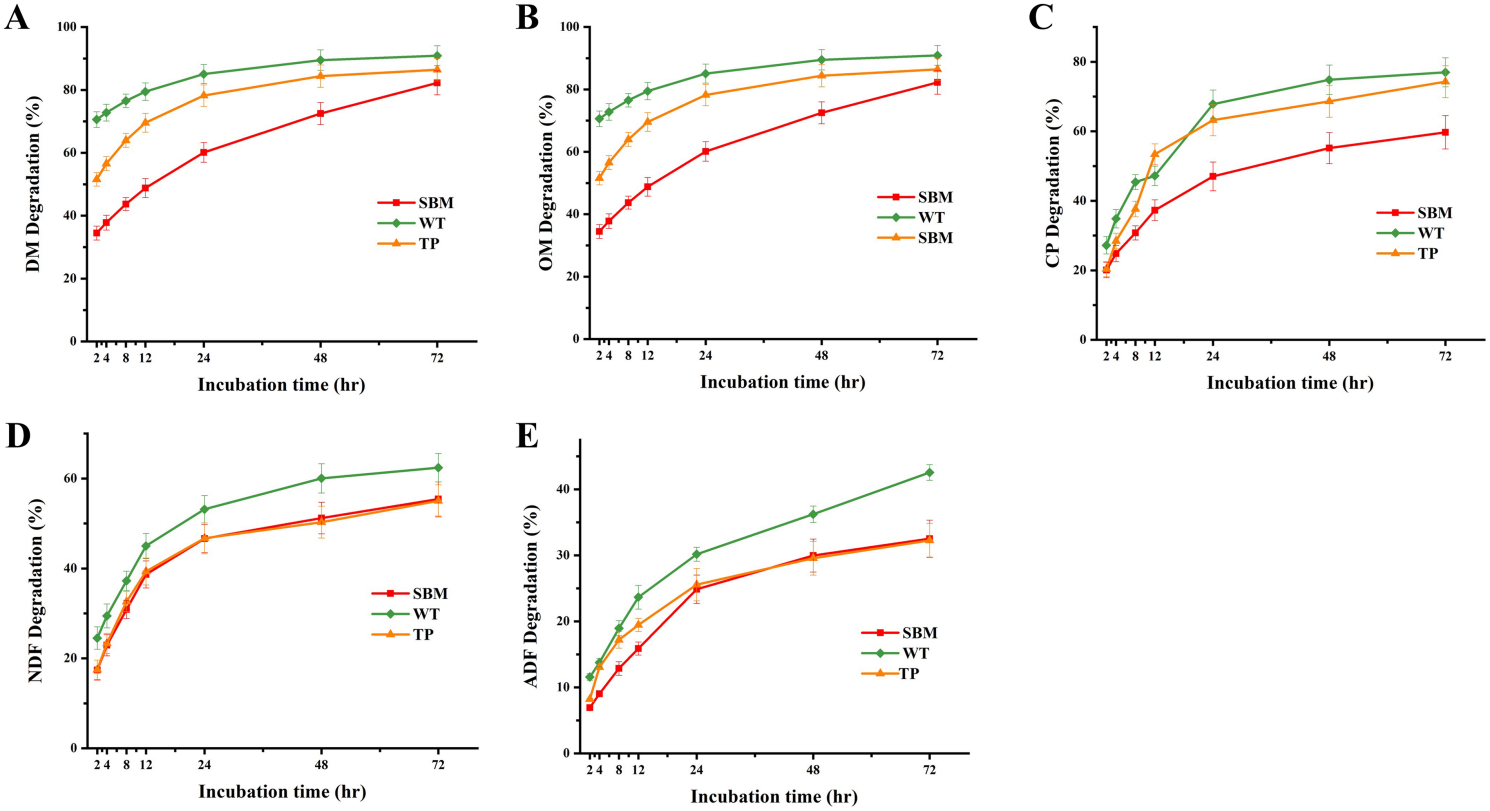
Trend diagram of the degradation curve of nutritional parameters for different feed materials incubated *in situ* in the rumen over various time periods. **(A)** DM degradation trend; **(B)** OM degradation trend; **(C)** CP degradation trend; **(D)** NDF degradation trend; **(E)** ADF degradation trend.

As shown in Figure 1, it can be intuitively concluded that the degradation rates of DM, OM, CP, NDF, and ADF in WT are higher than those in TP and SBM. Furthermore, the degradation rates of DM, OM, and CP in TP were lower than those in WT but higher than those in SBM. Finally, the degradation rate trends of NDF and ADF in TP were similar to those in SBM.

### 3.9 Effect of different treatments on Idg and IDCP of RUP

In Table 10, WT and TP had higher (*p* < 0.01) Idg and IDCP parameter values than SBM. Among them, WT had the highest (*p* < 0.01) IDCP parameter value, which was significantly greater than that of any other group.

**Table 10:** Effect of different treatments on Idg and IDCP of RUP.

Items	SBM	WT	TP	SEM	*p*-value
Idg, %	71.24^b^	79.50^a^	76.97^a^	0.75	<0.01
IDCP, g/kg	555.33^c^	674.58^a^	622.90^b^	10.67	<0.01

Idg, small intestinal digestibility of CP, IDCP, small intestine digests CP.Different superscript letters within the same row indicate significant differences in the data for this parameter (*p* < 0.05).

### 3.10 Total biodegradation rate of CP in different feed ingredients

**Figure 2:**
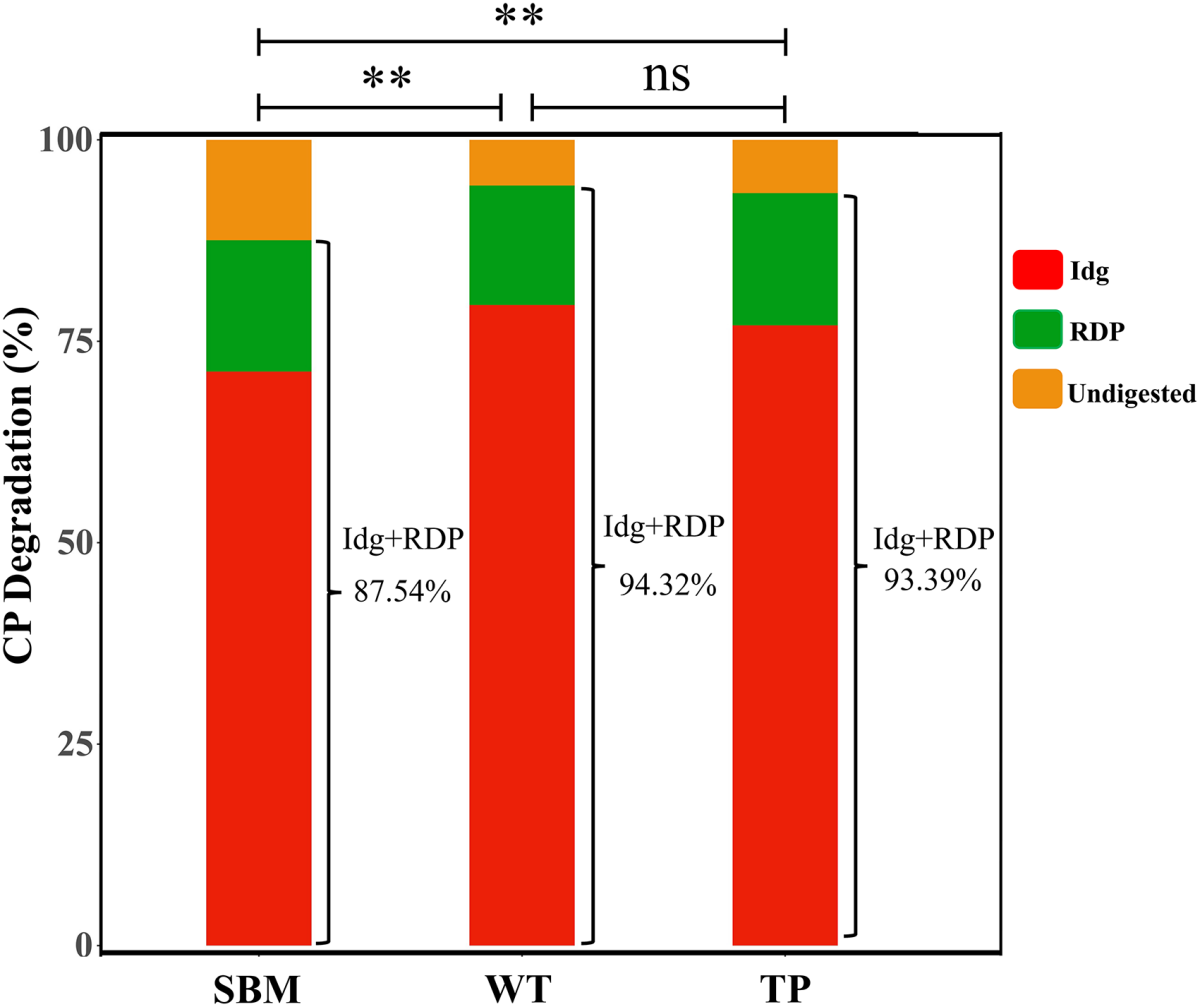
Total biodegradation rate of CP in different feed ingredients (SBM, WT, and TP). IDG + RDP, total biodegradation rate. ^*^p < 0.05; ^**^p < 0.01; ns, no statistical significance.

In Figure 2, the total biodegradation rate of SBM, WT, and TP was 87.54, 94.32, and 93.39%, respectively. Compared with SBM, the total biological degradability of CP in both WT and TP was increased (*p* < 0.01), while the total biodegradability rate of CP in WT and TP was not statistically significant (*p* > 0.05).

## 4 Discussion

### 4.1 Chemical composition of TP

TP, a by-product generated during tomato processing, predominantly consists of tomato seeds (65–72%) and peels (28–35%) (2). Del Valle et al. (6) documented that the nutritional composition of TP includes crude protein (CP: 15.4–23.7%), ether extract (EE: 5.4–20.5%), and fiber components (25.4–50.0% dry matter basis). Existing studies (2, 16, 32) have established that the nutritional constituents of TP—particularly CP, EE, NDF, and ADF—are principally derived from its seed fraction. In this study, our analysis confirmed that the CP and EE contents of TP fell within these ranges. Moreover, the CP, EE, NDF, and ADF levels in TP were relatively higher compared to WT. Of particular interest is the low acid detergent ADL content observed in TP, a finding that corroborates Fondevila et al. (33). This suggests TP may provide a higher proportion of digestible nutrients.

Tomatoes are primarily composed of the fatty acids C18:2n6, C16:0, and C18:3n3 (34, 35), which were similar to our results. Lu et al. (16) documented that the contents of SFA, MUFA, n-6 PUFA, and n-3 PUFA in TP ranged from 190.0 to 322.2 g/kg, 110.0–207.9 g/kg, 398.6–530.7 g/kg, and 42.2–156.6 g/kg, respectively. Of particular metabolic significance are the n-3 series fatty acids, specifically C20:3n-3 and C18:3n-3, which play essential roles in human physiology (36, 37). This study revealed that the n-3/n-6 ratio in TP was lower than in WT. Thus, based solely on the fatty acid composition, we believe TP may be a healthy source of feed raw materials. To our knowledge, the amino acid composition of TP is mainly composed of glutamic acid, aspartic acid, and leucine (2, 38). In addition, Elbadrawy and Sello (1) concluded that glutamic acid is the main amino acid in tomato peels. However, the amino acid content in tomato seeds is usually higher than that in tomato peels; therefore, the amino acid composition of TP depends mainly on the seed/peel ratio (38, 39). The amino acid concentration in TP was higher than in WT in this study, primarily because TP is richer in seeds and has a higher seed/peel ratio, as confirmed by previous studies.

### 4.2 Rumen *in situ* incubation and *in vitro* intestinal digestibility of CP

In this study, we found that the ED of DM, OM, CP, NDF, and ADF of WT and TP were higher than those of SBM, while the ED of WT was the highest, higher than that of TP. This is because WT and TP contain higher levels of water-soluble sugars, fruit acids, and other soluble substances compared to SBM, which are easily and rapidly degraded by microorganisms in the rumen. As a result, WT and TP exhibit higher solubility, leading to the higher a^*^ values observed in this study (2, 34, 40). Additionally, it was also demonstrated by Abbeddou et al. (41) that the non-structural carbohydrates and CP of TP were easy to degrade and degraded rapidly, which is an important factor for the higher degradation rates of WT and TP in this study. Notably, ADL plays a significant role in limiting NDF degradability, with ADL content being inversely correlated with NDF degradation (42). Nevertheless, the lower ADL content in WT and TP may suggest a greater proportion of digestible components (33, 57). Therefore, one potential reason for the higher degradation rates of other nutrients in this study could be the lower ADL content in WT and TP.

On the other hand, we propose that the higher degradation rates of WT and TP may also stem from tomatoes’ richness in bioactive compounds (e.g., lycopene, carotene, vitamins, and soluble dietary fiber) (43–45). Lycopene, an acyclic isomer of β-carotene, exhibits strong antioxidant properties and may enhance the activity of digestive enzymes (e.g., protease, cellulase, amylase) and antioxidant-related enzymes, thereby improving feed degradation (2, 46, 47). Among all bioactive compounds, lycopene occurs in the highest concentrations in WT and TP (48). Our analysis revealed lycopene concentrations of 75.98 μg/g (WT) and 63.61 μg/g (TP). Although these values were lower than those reported by Shao et al. (98.16–172.07 mg/kg) (49), they fell within the range of earlier studies (55.6–169 mg/kg) (50).

Notably, the lycopene content in WT was greater than in TP, suggesting that tomato pulp has a higher lycopene content compared to the peels and seeds. A previous scholar also indicated that the lycopene content in tomato seeds was much lower than that in tomato peels (39). Unfortunately, a separate analysis of the seeds and peels was not conducted in this study, which is an obvious limitation of this study. Numerous studies have demonstrated that the incorporation of lycopene in the diets of animals can improve the digestibility of nutrients (21, 46, 51, 52). Together, these factors likely explain the superior degradation rates of WT and TP over SBM. However, further in-depth studies are needed to explore the long-term effects of TP and lycopene supplementation in goats.

In this study, we found that the Idg and IDCP values of WT and TP were significantly higher than those of SBM, indicating that more than half of the CP undegraded in the rumen could be digested in the small intestine, a result higher than previously reported by other researchers (32, 33). Moreover, it has previously been suggested that the intestinal digestibility of CP is inversely proportional to its ruminal degradation rate (53); however, our study did not yield similar results. We believe this is attributable to the unique protein matrix or fiber composition of TP.

Additionally, differences may be related to variations in the *in situ* procedure (including animal species, diet formulation, and incubation protocols) and the rearing environment of goats (54). The primary reasons for the lower ruminal degradation rate and intestinal CP digestibility of SBM relative to WT and TP could be SBM’s higher protein content, lower soluble carbohydrate levels, more complex protein structure, and potentially higher levels of anti-nutritional factors, all of which contribute to slower degradation rates in both the rumen and the intestine (31, 55, 56). We also observed that the total CP biodegradation rate of WT and TP was higher than that of SBM. Based on these results, we conclude that WT and TP are more easily degraded than SBM.

## 5 Conclusion

Under the conditions of this study, most of the degradation kinetics parameters of DM, OM, CP, NDF and ADF of WT and TP were higher than those of SBM. Furthermore, compared with SBM, the CP of WT and TP were also easier to absorb in the intestine. In conclusion, based on the results of this study, although TP shows considerable promise, larger-scale *in vivo* studies are required to validate these findings.

## Data Availability

The original contributions presented in the study are included in the article/supplementary material, further inquiries can be directed to the corresponding author.
